# Lab-scale preparation and QC of phytase assay substrate from rice bran

**DOI:** 10.1016/j.ab.2019.04.021

**Published:** 2019-08-01

**Authors:** Claus Krogh Madsen, Charles Alistair Brearley, Henrik Brinch-Pedersen

**Affiliations:** a Institute of Molecular Biology and Genetics, Aarhus University, Forsogsvej 1, 4200, Slagelse, Denmark; b School of Biological Sciences, University of East Anglia, Norwich Research Park, Norwich, NR4 7TJ, UK

## Abstract

Phytases are involved in the phosphate acquisition and remobilization in plants, microbes and animals. They have become important technical enzymes in the feed industry and are used to make phosphate, present in animal feed as phytate, available for monogastric animal nutrition. Phytases may also be beneficial to human nutrition because phytate is known to interfere with the uptake of important micronutrients. Accordingly, phytases attract considerable research attention and phytate substrate lacking contaminants that interfere with commonly used phosphate-release assays is essential for this field of science. A procedure to prepare suitable sodium phytate from rice bran is presented. Extracted phytate is precipitated with barium hydroxide and re-dissolved in methanol after washing steps and sulphuric acid treatment. Remaining impurities are precipitated before the dissolved phytate is recovered as the sodium salt by addition of sodium hydroxide. In order to make the substrate widely available for research communities, the procedure relies solely on basic laboratory equipment and materials. Methods for quality control and monitoring of the purified sodium phytate or commercial alternatives are also presented.

## Highlights

• Sodium phytate suitable for assays was prepared from rice bran.
• The purification require no special equipment and can be performed in most biochemistry labs.
• Directions are given for quality control of the product.

## 1 Introduction

Phytases (myo-inositol hexakisphosphate 3-, 5- and 6-phosphohydrolase; EC 3.1.3.8, EC 3.1.3.72 and EC 3.1.3.26) are phosphatases that can initiate the stepwise hydrolysis of phytate (InsP_6_, myo-inositol-(1,2,3,4,5,6)-hexakisphosphate) [1,2]. Microbial phytases are important technical enzymes in the feed industry and plant phytases are attracting research attention for their importance in plant adaptation as well as their positive effects in human and animal nutrition [[3], [4], [5]]. The ability to accurately assay phytase activity is critical for these research efforts. Phytase assays measure the release of free phosphate from phytate, usually in the form of dodecasodium phytate e.g. as described by Engelen [6]. Phytate is purified from plant seeds and may vary in quality depending on origin and purification procedure. Certain common contaminants and shortcomings are particularly detrimental to phytase assays. They are: 1) free phosphate (Pi), since it gives background absorbance which limits the accurate range of the assay, and potentially inhibit phytases via product inhibition; 2) Lower inositol phosphates (InsP_x<6_), since they are substrates of phosphatases which are not phytases; contamination with lower inositol phosphates can lead to misclassification of novel enzymes and overestimation of phytase activity in complex samples; and 3) Inaccurate sodium content, contaminating ions and moisture as this directly influences the molecular weight of the substrate and therefore leads to the use of inaccurate substrate concentrations. Some contaminating ions may even act as inhibitors or enhancers of the phytase being assayed. Furthermore, phytate for routine assays should be reasonably priced. Until now, our lab has used Sigma P0109 or P3168 phytate as substrate in our phytase assays. Unfortunately, these products are not available anymore and we and other labs have faced severe difficulties in finding a suitable replacement from a commercial supplier. Current commercially available products suffer from at least one of the quality shortcomings mentioned above and compromise the quality of the phytase activity assay. In order to continue our research on phytases, we found it necessary to develop a protocol for purification of quality phytate to be used for phytase assays. Moreover, we aimed for developing a protocol without chromatographic steps that in most labs would limit the batch size or make the procedure unavailable to laboratories without specialized equipment. First we developed a procedure for recrystallizing commercially available sodium phytate, thereby removing free phosphate and adjusting the sodium content. However, this proved to be insufficient for achieving the necessary purity. Subsequently we have developed a procedure to purify phytate directly from rice bran, a cheap byproduct from polishing of rice that is available in large quantities. Procedures to verify the quality of the substrate are also described.

## 2 Materials and methods

### 2.1 Materials

Sodium phytate P0109-100 g lot#057K0049 (discontinued), sodium phytate P8810-500 g Lot#BCBQ7037V, sweet potato acid phosphatase P1435–500UN and *A. ficuum* phytase P-9792 were purchased from Sigma-Aldrich (now Merck), Darmstadt, Germany. Pelleted rice bran was purchased from Mühldorfer Pferdefutter, Mühldorf, Germany. Wheat (*Triticum aestivum* L.) cv. Skagen and Rye (*Secale cereale* L.) cv. Picasso were field grown in Denmark. Whole grain basmati type rice (*Oryza sativa* L.) was purchased from a local supermarket. Broad bean (*Vicia faba* L.) cv. Hangdown and Soy bean (*Glycine* max L.) cv. Fiskeby V were purchased from Weibulls, Åby, Sweden trough a local garden center.

### 2.2 Recrystallization of sodium phytate

A detailed protocol for recrystallization is found in appendix A. Briefly, sodium phytate is dissolved in 1 M hydrochloric acid, adjusted to pH 0.5 and precipitated by the addition of methanol to 70%. The precipitate is re-dissolved in 1 M sodium hydroxide and adjusted to pH 11. Addition of methanol to 10% precipitates impurities. Sodium phytate is recovered by increasing the methanol concentration to 80%. The precipitate is washed in methanol and lyophilized.

### 2.3 Purification of sodium phytate from rice bran

A detailed protocol for purification is found in appendix B. Briefly, rice bran is defatted with acetone and phytate is extracted with 0.25 M hydrochloric acid. The extract is clarified and phytate is precipitated with barium hydroxide at pH 5. The crude barium phytate is washed with water and 0.1 M sodium hydroxide with 0.01 M EDTA to remove co-precipitating cations. The barium phytate is converted to barium sulfate and free phytic acid with 10 M sulphuric acid. Phytic acid is extracted from the mixture with methanol and precipitated as sodium phytate by addition of 1 M sodium hydroxide. The precipitate is washed with 75% methanol, re-dissolved in water and adjusted to pH 12.3. Impurities are precipitated and sodium phytate is recovered as described above.

### 2.4 Phytase assays were performed as described by Engelen et al. [6]

#### 2.4.1 Quality control was performed according to appendix C

Briefly; pH was measured according to Evans [7] with a Biotrode pH electrode (Metrohm). Magnesium was assayed with azoviolet. The phytase assay was modified for quality control by recording and comparing absorbance values for A) Blank, buffer and stop solution only. B) P_i_ background, substrate solution and stop solution subtracted blank. C); InsP_x<6_, substrate solution incubated with 0.2 units sweet potato phosphatase before adding stop solution subtracted blank + Pi background; D) Phytate, substrate solution incubated with 3.5 × 10^−2^ units *Aspergillus ficuum* phytase before adding stop solution subtracted blank + Pi background.

### 2.5 HPLC analysis

Inositol phosphates were separated by anion exchange HPLC on a 3  mm × 250  mm CarboPac PA200 column (Dionex, Sunnyvale, CA) fitted with a 3  mm × 50  mm guard column of the same material. The column was eluted with a gradient of methanesulfonic acid delivered at a flow rate of 0.4 mL min^−1^ by mixing of solvents from reservoirs containing water (A) and 0.6 M methanesulfonic acid (Acros Organics) (B) according to the schedule: time (min), %B; 0,0; 25,100; 38,100; 39,0; 49,0. The column eluate was mixed in a mixing Tee with a solution of 2% w/v ferric nitrate (nonahydrate) in 0.1% v/v perchloric acid delivered at a flow rate of 0.2 mL min^−1^. The combined flow was passed through a 4  m × 0.25  mm i.d. knitted reaction coil (Biotech AB, Sweden) and inositol phosphate peaks were monitored by UV absorbance at 290 nm [8]. Peak areas were integrated with ChromNav (Jasco) software and compared to that of standards of Na_12_InsP6 (Merck Millipore – Calbiochem Cat: 407125-25 MG Lot: 2663470). The Jasco LC-4000 HPLC system comprised: an AS-40140 autosampler, PU-4085i and PU4080i pumps, a UV-4070 UV-visible detector and a CO-4061 column oven.

An acid-hydrolysate of phytate was obtained by refluxing 3 g sodium phytate (Sigma P8810) in 100 mL of 1 M HCl for 24 h. The resulting solution was lyophilized to near dryness, made up to 100 mL with water, lyophilized again and made to a final volume of 100 mL with water. For use as an HPLC standard, the hydrolysate was diluted 20-fold with water and 20 μL injected onto HPLC. For reproduction of HPLC profiles, data was exported from ChromNav as an ASCII file and redrawn in GraFit v.7 [9].

### 2.6 Elemental analysis

Elemental analysis was done by ICP-SFMS in compliance with SS EN ISO 17294-1, 2 (mod) and EPA-method 200.8 (mod) by ALS Scandinavia AB, Luleå, Sweden.

## 3 Results and discussion

**Fig. 1:**
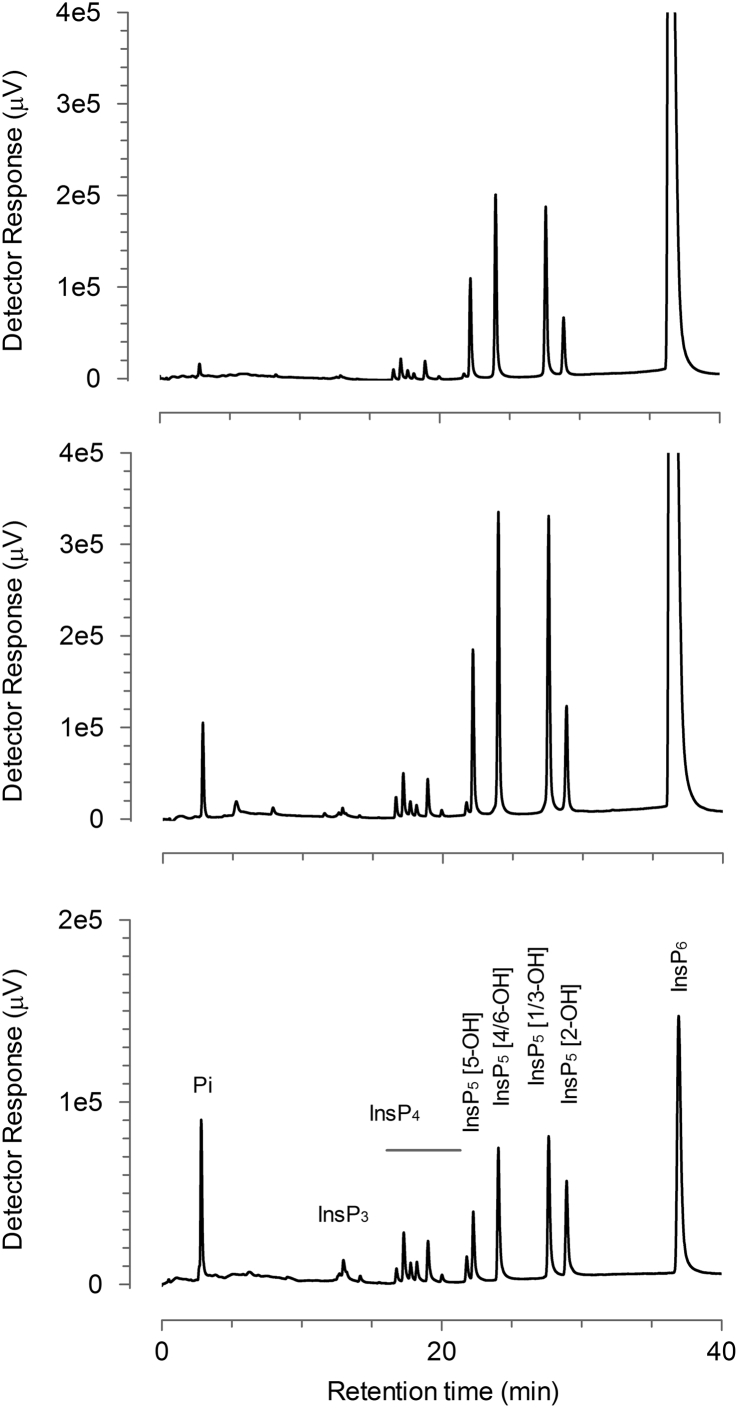
HPLC chromatograms of P8810 RC (top), original P8810 sodium phytate (middle) and an acid- hydrolysate of InsP__6__ with InsP__x__ species identified.

**Fig. 2:**
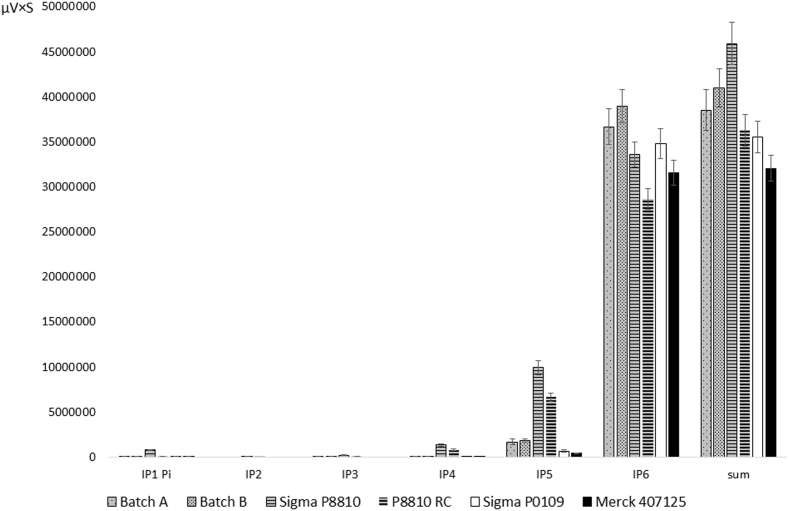
Peak areas from HPLC profiling. Error bars show the standard deviation of triplicate runs.

Sodium phytate P8810 has been suggested as substitute for the discontinued P0109/P3168 but contains unacceptable levels of Pi that results in a background absorbance of >1 in the phytase assay. It was also noticed that P8810 produced a much lower pH value in solution than P0109 (Table 1). Preliminary experiments showed that phytate precipitates well in methanol >70% whereas ethanol forms a two phase system (results not shown). It was hypothesized that a double re-crystallization in methanol would render Pi contaminated products suitable for phytase assays. The first re-crystallization was done below the most acidic pKa of phytate (as reported by Evans [7]) to remove metallic cations as much as possible. The second recrystallization was done after adjusting pH above the highest pKa with sodium hydroxide to obtain the dodecasodium salt. A reddish brown discoloration appeared after the addition of sodium hydroxide. This could be precipitated by the addition of methanol to 10% before the final precipitation in 80% methanol. The yield of re-crystalized P8810 (P8810 RC) was 7.7 g using 10 g P8810 as starting material. Re-crystallization greatly reduced Pi and increased the pH in solution (Table 1). Hydrolysis of phytate to InsP_x<6_ is the likely source of Pi contamination. Sweet potato acid phosphatase was used to screen the re-crystalized product for InsP_x<6._ It was evident that the re-crystalized P8810 was more prone to hydrolysis by acid phosphatase than P0109 (Table 1). HPLC profiling confirmed that P8810 and re-crystalized P8810 contained >20% lower inositol phosphates and the inositol phosphate profile was affected very little by the recrystallization (Fig. 1, Fig. 2 and Table 2). Since InsP_x<6_ are difficult to separate from phytate on a preparative scale it was decided to attempt purification directly from rice bran.

**Table 1:** Assay absorbance and pH values measured by the quality control procedures. SD denote standard deviation of triplicate repetitions.

	P_i_		IP_x<6_		Phytate		pH	Mg test
	Abs 415 nm	SD	Abs 415 nm	SD	Abs 415 nm	SD		Abs 595/555 nm
Sigma P0109	0.084	1.52 × 10^−3^	0.060	2.31 × 10^−3^	0.817	13.7 × 10^−3^	10.15	0.71
Sigma P8810	1.195	1.53 × 10^−3^	0.493	42.5 × 10^−3^	0.930	13.9 × 10^−3^	4.21	0.71
P8810 RC	0.159	2.31 × 10^−3^	0.395	1.53 × 10^−3^	0.932	19.1 × 10^−3^	10.00	0.81
Purified from rice bran, batch A	0.100	3.2 × 10^−3^	0.052	5.51 × 10^−3^	0.993	20.1 × 10^−3^	9.78	1.39
Purified from rice bran, batch B	0.092	18.9 × 10^−3^	0.107	15.9 × 10^−3^	0.834	27.6 × 10^−3^	10.15	0.72

**Table 2:** Peak areas from the HPLC profiling given as percentage of the total peak area. Values after ± show the standard deviation of triplicate runs.

	InsP1, Pi	InsP2	InsP3	InsP4	InsP5	InsP6
Batch A	0.23 ± 0.03	0.00 ± 0.00	0.13 ± 0.04	0.11 ± 0.05	4.38 ± 0.51	95.18 ± 0.49
Batch B	0.11 ± 0.03	0.00 ± 0.00	0.24 ± 0.01	0.10 ± 0.03	4.48 ± 0.32	95.08 ± 0.37
Sigma P0109	0.13 ± 0.00	0.00 ± 0.00	0.00 ± 0.00	0.02 ± 0.00	1.90 ± 0.21	97.96 ± 0.21
Sigma P8810	1.71 ± 0.12	0.12 ± 0.02	0.45 ± 0.04	2.90 ± 0.15	21.62 ± 0.59	73.19 ± 0.67
P8810 RC	0.24 ± 0.03	0.01 ± 0.01	0.21 ± 0.08	2.21 ± 0.09	18.37 ± 0.40	78.96 ± 0.56
Merck 407125	0.15 ± 0.03	0.00 ± 0.00	0.00 ± 0.00	0.03 ± 0.03	1.34 ± 0.13	98.48 ± 0.18

The procedure builds on the re-crystallization protocol but introduces a series of preceding steps. A) Acidic extraction, this solubilizes phytate and is commonly used in analytical protocols [10]. B) Precipitation with Ba^2+^, barium phytate precipitates at lower pH than e.g. calcium phytate [11]. The content of contaminating ions can therefore be reduced at an early stage by controlling pH. Some cations such as proteins with pI > 5 co-precipitate [12]. An alkaline wash was included to remove these impurities from the precipitate. C) Removal of barium with sulphuric acid, this step precipitates barium as the insoluble sulfate. Phytate was readily soluble in methanol after this conversion and could be extracted from the barium sulfate pellet. Addition of sodium hydroxide precipitates phytate and the procedure continues essentially as the re-crystallization. To our knowledge, this is the first report of a procedure that uses conversion of barium phytate with sulphuric acid to produce sodium phytate.

A batch (A) was produced from 100 g defatted rice bran and precipitated from pH 11 in the final step. The yield was 2.1 g. The Pi background and resistance to hydrolysis by acid phosphatase (InsP_x<6_) of the product were in the same range as P0109 but the absorbance produced with phytase was approximately 22% higher (Table 1). A higher phytase assay absorbance may be caused by insufficient sodium content but the pH of the product in solution was above the pKa value (9.5) of the 12'th titrateable H reported by Evans et al. [7]. Elemental analysis of the batch revealed that it was essentially 82% magnesium decasodium phytate (Table 3). Presumably, magnesium remains associated with phytate throughout the procedure because it forms a soluble complex with phytate below pH 5 and coprecipitates as a mixed salt when barium is added [11]. The difference in molecular weight between the MgNa_10_ and Na_12_ phytates does not offer a satisfying explanation for the differences in phytase assay absorbance. More likely, it is the stimulatory effect of Mg^2+^ on *Aspergillus* phytase [13].

**Table 3:** Elemental analysis. CHO was calculated based on the P content by assuming InsP6. Stoichiometric coefficients marked with * are theoretical.

Element	Batch A		Batch B	
	Content (mg/g)	Stoichiometric coeff.	Content (mg/g)	Stoichiometric coeff.
P	170	6.0*	166	6.0*
Na	203	9.7	212	10.3
Mg	20.6	0.93	0.121	5.57 × 10-3
C_calc_	65.9	6.0*	64.4	6.0*
O_calc_	351	24*	343	24*
H_calc_	5.54	6.0*	5.41	6*
Sum of the above	816.0		790	
Others total	3.2		4.9	

The procedure was modified by increasing the pH before the precipitation of impurities to 12.3 to precipitate magnesium as hydroxide and a qualitative test for magnesium was included in the quality control. The test utilizes the shift in azoviolet absorbance in the presence of Mg^2+^ at alkaline pH. An additional batch (B) was produced by this procedure yielding 2.3 g which did not respond to the azoviolet test. Elemental analysis confirmed that this batch contains minimal amounts of Mg and appears to consist of 79% decasodium phytate (Table 3). Other elements included in the elemental analysis accounted for 0.5% leaving 20.5% of the mass unexplained. Batch A and B were HPLC profiled together with original and recrystallized Sigma P8810, the discontinued Sigma P0109 and a dodecasodium phytate standard (Fig. 2, Table 2). This revealed that 95% of the detected peak area in Batch A and B was InsP6. InsP5 was the major contaminant, making up 4–5% of the samples. The discontinued Sigma P0109 and the standard were both approximately 98% InsP6 whereas Sigma P8810 was only 73%.

Since the elemental analysis included the most likely cationic contaminants such as barium, iron and calcium and the HPLC analysis further confirmed the purity of batch A and B, we suggest that our product contains approximately 20% w/w crystal water. Nevertheless, they have the largest InsP6 content. The commercial products P0109, P8810 and P3168 also contained an unspecified amount of crystal water according to manufacturer (products were labeled x or yH_2_O). Engelen et al. [6] gave the formula C_6_H_6_Na_12_O_24_P_6_ × 10H_2_O with reference to P3168. This corresponds to approximately 17% w/w crystal water. Thus we conclude that our product is not different from available and discontinued commercial products with respect to having a substantial amount of crystal water. Our product and Sigma P0109 produced the same pH in solution, suggesting that both are in fact decasodium phytate rather than dodecasodium phytate. This may also contribute to the larger peak area of Sigma P0109 compared to the dodecasodium phytate standard. The molecular weights of the anhydrous deca- and dodeca sodium phytates are 877.83 and 923.81 g/moles respectively so the decasodium salt would produce a 5% larger peak (observed 10%).

A phytase assay was performed to compare the performance of P0109, recrystallized P8810 and batch B as substrate for the phytase activities of complex samples. Wheat, rye, rice, soy bean and broad bean were chosen as known high and low phytase materials, representing both monocots and dicots. The assay did not reveal any difference between the three substrates (Table 4).

**Table 4:** Phytase assay results of three substrates on five complex samples. Values are in FTU/Kg (1 FTU liberates 1 μmole P_i_ from sodium phytate per minute at 37ᵒC and pH 5.5) and ± denotes the standard deviation of triplicate samples.

	Rice Basmati	Rye cv. Picasso	Wheat cv. Skagen	Soybean cv. Fiskeby V	Broard bean cv. Hangdown
Sigma P0109	131.9 ± 160.7	3470.7 ± 160.0	1215.2 ± 129.2	−271.9 ± 153.0	−25.6 ± 203.7
P8810 RC	263.3 ± 31.6	3648.5 ± 176.8	1383.7 ± 147.9	−34.8 ± 22.5	−34.8 ± 39.4
Batch B	202.2 ± 30.6	3328.1 ± 138.8	1189.3 ± 75.2	−207.0 ± 17.0	2.2 ± 3.2

## 4 Conclusion

Using feed grade rice bran and simple chemicals and equipment, we were able to produce high quality decasodium phytate in >2 g batches. The two batches presented here are almost identical with respect to the distribution of inositol phosphates and yield. Only the occurrence of magnesium was different between the batches. The final adjustments to the protocol solved the problem with magnesium and the proposed quality control consider this risk. Earlier batches (not reported) were also consistent with respect to yield and lower inositol phosphates so the procedure is reproducible. It should be noted that the content of lower inositol phosphates is influenced by the quality of the starting material. Rice bran that has been exposed to excessive heat or moisture during storage should be avoided. Batch B was almost equivalent to the discontinued Sigma P0109 on the critical parameters of P_i_ background, contamination with lower inositol phosphates and sodium content (as judged by pH in solution). A batch at this size is sufficient for approximately 600 phytase assay reactions. It is possible to verify the quality of the product in any lab equipped for phytase assays with an enzymatic assay, a colorimetric test for magnesium and pH measurements. The procedure therefore provides an open and independent source of phytase substrate for the research community.

Re-crystallization of commercially available sodium phytate may be sufficient for some applications e.g. screening for variation in the phytase activity of complex samples with a low phosphatase background. However, characterization of novel purified enzymes and phytase assays in a high phosphatase background requires sodium phytate with minimal amounts of lower inositol phosphates.

We urge researchers in the field to verify the quality of their phytase substrate for instance using the methods described here.

## Appendix A

### Recrystalization of sodium phytate

#### Safety

Methanol is toxic and flammable. Sodium hydroxide and hydrochloric acid are corrosive. Observe MSDS and local regulations.

#### Acidic recrystallization

Dissolve 10 g sodium phytate in 15 mL 1 M hydrochloric acid. Adjust the pH to 0.5 with 6 M hydrochloric acid. Measure the volume with a pipette or measuring glass. Distribute maximum 15 mL in 50 mL centrifuge tubes. Add methanol to a final concentration of 70%. Place on ice for 10 min. Centrifuge at >7100×*g* at 4 °C for 10 min. Decant the supernatant. Centrifuge again briefly to remove all of the supernatant. Allow the remaining methanol to evaporate by leaving the tubes open in the fumehood for minimum 2 h. The pellets need not be dry.

#### Precipitation of impurities

Redissolve the pellets in a 25 mL (total volume) 1 M sodium hydroxide. Adjust the pH to 11 with 5 M sodium hydroxide. Note that the solution loose buffer capacity above pH 10.

Add three volumes 13.3% methanol. Centrifuge at >7100×*g* at 4 °C for 10 min. A small redish brown pellet may form.

- If an aqueous two phase system is formed, dilute with 10% methanol until it disappears.
- If a large pellet is formed (more than 0.5 mL per 50 mL), dilute as above.

Centrifuge again after dilution and collect the supernatant.

#### Alkaline precipitation

Increase the methanol concentration to 80%. Place on ice for 1 h or at 4 °C over night. Isolate the precipitate by centrifugation at >7100×*g* at 4 °C for 45 min. Wash the pellets in approximately 3 volumes methanol. Remove the methanol after a brief centrifugation. Lyophilize the pellets and pulverize in a ceramic mortar.

## Appendix B

### Preparation of sodium phytate from rice bran

#### Safety

Methanol and barium hydroxide are toxic substances. Methanol and acetone are flammable. Sodium hydroxide, hydrochloric acid and sulphuric acid are corrosive. Observe MSDS and local regulations.

#### Defatting

Cover rice bran with acetone (approximately 1 mL/g). Leave for at least 4 h to extract. Decant the acetone and add fresh acetone. Leave overnight, filter and air-dry the bran overnight in the fume hood.

#### Hydrochloric acid extraction

Place 100 g of defatted rice bran in a beaker and add 500 mL 0.25 M hydrochloric acid (e.g. 20.8 mL 6 M HCl+479.2 mL water). Stir vigorously for 1 h. Centrifuge at > 7100 × g for 10 min and collect the supernatant. Vacuumfilter the supernatant trough a filter paper to remove remaining debris.

#### Precipitation of barium phytate

Place the hydrochloric extract in a beaker with stirring and pH monitoring. Add 5 g barium hydroxide. Leave with stirring until the powder has completely dissolved and a new precipitate is starting to form. Adjust the pH to 5.0 with 5 M sodium hydroxide to complete precipitation. Centrifuge at >7100 × g for 5 min and discard the supernatant. Wash the precipitate in the following sequence: once with demineralized water, two times with 0.1 M sodium hydroxide with 0.01 M EDTA, and finally once with water. For each wash, use 180 mL (30 mL per tube when using six tubes), carefully resuspend and spin down at >7100 × g for 5 min (10 min for the final wash).

#### Conversion to barium sulfate and phytic acid

Weigh the wet precipitate in the tubes. Expect around 10–15 g total. Place the tubes on ice and add 160 μL 10 M sulphuric acid per gram wet precipitate. Immediately mix with a glass rod to obtain a homogenous suspension. Proceed when the precipitate has been completely re-suspended but do not exceed 30 min.

**First crystallization:** Add 100 mL methanol (16.7 mL per tube if six tubes are used). Shake and vortex to resuspend, then spin down at >7100 × g for 5 min. Collect the supernatant in a 250 mL flask. Avoid carryover of the pellet (e.g. use a paper filter). Distribute the liquid evenly in three 50 mL centrifuge tubes. Add 13.3 mL 1 M sodium hydroxide per tube. Vortex or shake and allow the precipitation to proceed for minimum 5 min. Spin down the precipitate at >7100 × g for 5 min. Wash the precipitate with 20 mL 75% methanol per tube (stopping point – the precipitate may be stored refrigerated in the 75% methanol overnight or longer before proceeding). Air-dry the precipitate for minimum 2 h in a beaker.

**Re-dissolve:** Add 50 mL of water and stir to re-dissolve the precipitate. Adjust the pH to 12.3 with 5 M sodium hydroxide. Stop the titration if the pH begin to fall at approximately the target pH. Finally adjust the volume to 90 mL.

#### Precipitate impurities

Add 10 mL methanol while stirring. If a two phase system forms, dilute with 10% methanol until the phases merge. Centrifuge at >7100 × g for 10 min and collect the supernatant.

#### Final precipitation

Increase the methanol concentration to 80%. Place on ice for minimum 15 min. Collect the precipitate by centrifugation at >7100 × g at 4 °C for 10 min. Wash the precipitate in 60 mL 75% methanol followed by 120 mL methanol. Lyophilize the precipitate for minimum 36 h. Do not dry by heating. Pulverize the dried material in a ceramic mortar.

## Appendix C

### Quality control of sodium phytate

#### Part 1

A) Prepare a 0.01 M solution of the analyte (9.2 mg/mL) in demineralized water. Mix one part 4 M potassium chloride, two parts analyte solution and 17 parts demineralized water. Measure pH, Expect 10.15 for dodecasodium phytate. Higher values may be caused by residual sodium hydroxide. Lower values may be caused by insufficient sodium content or contaminating cations.
B) Prepare a solution of 1 mg/mL azoviolet (4-(4-Nitrophenylazo)resorcinol) in ethanol. Combine 100 μL analyte solution with 50 μL 1 M sodium hydroxide and 1 μL azoviolet solution in a microtitter plate. Scale up if conventional cuvettes are used. Measure the absorbance at 555 and 595 nm. The ratio 595/555 nm should be < 0.8. A higher ratio indicates contaminating magnesium. The shift in absorbance is visible to the naked eye as the color changes from violet to blue.

#### **Part 2**

A) Prepare the solutions for phytase assay.

**Assay buffer:** prepare 25 mM Sodium acetate pH 5.5 with 0.1 mM calcium chloride.

**Ammonium heptamolybdate stock:** dissolve 10 g ammonium heptamolybdate tetrahydrate in 90 mL 60 ᵒC water. Add 1 mL 25% ammonia and adjust the volume to 100 mL at room temperature with water. Store in the dark.

**Ammonium vanadate stock:** dissolve 235 mg ammonium vanadate in 40 mL 60 ᵒC water. While stirring slowly add 2 mL 23% nitric acid and adjust the volume to 100 mL at room temperature with water. Store in the dark.

**Color/stop mix** for 12 reactions: Combine 2.5 mL ammonium heptamolybdate stock with 2.5 mL ammonium vanadate stock, 1.65 mL 65% nitric acid and 3.35 mL water. Prepare fresh the same day as performing the assay.

**Substrate solution.** Dissolve 16.8 mg analyte dodecasodium phytate in 1 mL assay buffer. Adjust the pH to 5.5 with 4 M acetic acid. Finally adjust the volume to 2 mL with assay buffer.

**Enzyme solutions.** Prepare solutions of *Aspergillus ficuum* phytase, (Sigma P9792) 1 mg/mL in assay buffer and sweet potato phosphatase (Sigma P1435) 0.02 u/μL (dilute 1:49 in assay buffer).

B) Incubate samples at 37ᵒC for 60 min before adding color/stop mix. Centrifuge 5 min at 4000×*g* and measure the supernatant absorbance at 415 nm. Assay in triplicate and subtract the average of the three blanks form the absorbance values.

Blank: 800 μL assay buffer +800 μL color/stop mix.

Pi background: 400 μL assay buffer +400 μL substrate solution +800 μL color/stop mix.

IP_x<6_: 390 μL assay buffer +10 μL sweet potato phosphatase solution +400 μL substrate solution +800 μL color/stop mix.

IP6: 390 μL assay buffer +10 μL *Aspergillus ficuum* phytase solution +400 μL substrate solution +800 μL color/stop mix.

## References

[1] Suzuki, U. (1907) Uber ein Enzym "Phytase" das Anhydro-oxy-methylen-diphosphosaure spaltet. Bull. Coll. Agr. Tokyo Imper. Univ. 7, 503-505.

[2] Oh, B.-C. et al. (2004) Biochemical properties and substrate specificities of alkaline and histidine acid phytases. Applied Microbiology and Biotechnology 63 (4), 362-372. 10.1007/s00253-003-1345-0 14586576

[3] Brinch-Pedersen, H. et al. (2014) Increased understanding of the cereal phytase complement for better mineral bio-availability and resource management. Journal of Cereal Science 59 (3), 373-381.

[4] Lei, X.G. et al. (2013) Phytase, a New Life for an “Old” Enzyme. Annual Review of Animal Biosciences 1 (1), 283-309. 10.1146/annurev-animal-031412-103717 25387021

[5] Scholey, D. et al. (2017) P and Ca digestibility is increased in broiler diets supplemented with the high-phytase HIGHPHY wheat. Animal 11 (9), 1457-1463. 10.1017/S1751731117000544 28318476

[6] Engelen, A.J. et al. (1994) Simple and rapid determination of phytase activity. J AOAC Int 77 (3), 760-764. 8012231

[7] Evans, W.J. et al. (1982) Titration studies of phytic acid. Journal of the American Oil Chemists’ Society 59 (4), 189-191.

[8] Phillippy, B.Q. and Bland, J.M. (1988) Gradient ion chromatography of inositol phosphates. Analytical Biochemistry 175 (1), 162-166. 10.1016/0003-2697(88)90374-0 3245565

[9] Leatherbarrow, R.J. (2010), GraFit, Erithacus Software Ltd, Horley, U.K., http://www.erithacus.com/grafit/binary/grafit%20version%207.pdf.

[10] Victor, R. et al. (2017) Evaluation of Simple and Inexpensive High-Throughput Methods for Phytic Acid Determination. Journal of the American Oil Chemists' Society 94 (3), 353-362.

[11] Torres, J. et al. (2008) Interaction of myo-inositol hexakisphosphate with alkali and alkaline earth metal ions: Spectroscopic, potentiometric and theoretical studies. Journal of Molecular Structure 874 (1), 77-88.

[12] Selle, P.H. et al. (2012) Protein-phytate interactions in pig and poultry nutrition: a reappraisal. Nutrition Research Reviews 25 (1), 1-17. 10.1017/S0954422411000151 22309781

[13] Casey, A. and Walsh, G. (2003) Purification and characterization of extracellular phytase from Aspergillus niger ATCC 9142. Bioresource Technology 86 (2), 183-188. 10.1016/s0960-8524(02)00145-1 12653285

